# Overexpression of GATA5 Stimulates Paclitaxel to Inhibit Malignant Behaviors of Hepatocellular Carcinoma Cells

**DOI:** 10.22074/cellj.2020.6894

**Published:** 2020-09-08

**Authors:** Haipeng Feng, Bo Lin, Yifei Zheng, Junnv Xu, Ying Zhou, Kun Liu, Mingyue Zhu, Mengsen Li

**Affiliations:** 1. Hainan Provincial Key Laboratory of Carcinogenesis and Intervention, Hainan Medical College, Haikou, Hainan Province, China; 2. Department of Tumor Internal Medicine, Second affiliated Hospital of Hainan Medical College, Haikou, Hainan Province, China; 3. Institution of Tumor, Hainan Medical College, Haikou, Hainan Province, China

**Keywords:** GATA5, Hepatocellular Carcinoma Cells, Paclitaxel, Reprogramming Genes, Stemness Marker

## Abstract

**Objective:**

Explore the effect of GATA5 expression on Paclitaxel inhibiting growth of hepatocellular carcinoma (HCC) cells.

**Materials and Methods:**

In the experimental study, HCC cell lines (HLE, Bel7402 and PLC/PRF/5) were treated with different concentrations of Paclitaxel (5-20 mg/ml) for 24 hours. HLE cells were transfected with GATA5-siRNA vector, while Bel7402 and PLC/PRF/5 cells were transfected with overexpressed GATA5 vector for 24 hours, followed by treatment of the cells with Paclitaxel (10 mg/ml) for 24 hours and subsequently 3-(4,5-dimethyl-2-thiazolyl)-2,5-diphenyl-2-H-tetrazolium bromide (MTT) assay to detect growth of HCC cells. Soft agar cultured was used to analyze formation of colony. Apoptosis of HCC cells were detected by Flow cytometer. Migration of HCC cells was observed by trawell assays. Western blotting and laser confocal microscopy were utilized to detect expression and location of the proteins.

**Results:**

Inhibiting expression of GATA5 reduced sensitivity of HLE cells to Paclitaxel, while overexpression of GATA5 increased sensitivity of Bel7402 cells and PLC/PRF/5 cells to Paclitaxel. Overexpression of GATA5 played a role in stimulating Paclitaxel to inhibit growth, colony formation and migration, as well as enhance apoptosis in HCC cells. Overexpression of GATA5 also promoted Paclitaxel to inhibit expression of reprogramming genes, such as Nanog, EpCAM, c-Myc and Sox2 in Bel7402 and PLC/PRF/5 cells. Inhibited expression of GATA5 led to enhancement of the expression of CD44 and CD133, in HLE cells. Overexpression of GATA5 was not only alone but also synergized with Paclitaxel to inhibit expression of CD44 and CD133 in Bel7402 or PLC/PRF/5 cells.

**Conclusion:**

Overexpression of GATA5 played a role in enhancing Paclitaxel to inhibit the malignant behaviors of HCC cells. It was involved in suppressing expression of the reprogramming genes and stemness markers. Targeting GATA5 is an available strategy for applying paclitaxel to therapy of patients with HCC.

## Introduction

Paclitaxel is an effective chemotherapeutic drug that is widely applied in the treatment of a number of cancer types. It promotes cell death through apoptotic pathway (1, 2), while it causes drug resistance in the cancer cells (3). Hepatocellular carcinoma (HCC) is the fifth most frequent type of cancer and the rate of drug-resistance in HCC patients is high (3, 4). Surgery is considered as the best method for the treatment of liver cancer. However, many patients are diagnosed in the middle and late stages of disease and they loss chance of surgery. Thus, the mortality rate of liver cancer patients is higher than many other types of malignant tumor (3, 5). There is an imperative need to explore the mechanism of HCC cell resistance to drug therapy and to develop new strategy for treating drug-resistance of HCC patients.

GATA family regulates cell reprogramming to induce stem cell differentiation and normal function of cells (6). This family includes GATA1-6, while GATA3 plays a key role in the regulating breast cancer suppression (7). GATA5 also inhibits proliferation, invasion and migration of cholangiocarcinoma cells (8). Hypermethylation of gene promoter suppresses *GATA5* expression, leading to promoting growth and colony formation in HCC cells (9). Paclitaxel is a valid chemotherapy drug in HCC patients, although the corresponding drug-resistance has frequently been observed during treatment of these patients. GATA5 is an optional bio-target for treatment of HCC, however, the effect of *GATA5* expression on Paclitaxel during treatment of HCC patients is not clear yet.

Previously, evidences indicated high expression of some reprogramming genes and stemness markers in HCC cells (10-13). In this study, we investigated how GATA5 influenced proliferation, apoptosis, migration and invasion of HCC cells after treatment with Paclitaxel. The results displayed that overexpression of* GATA5* stimulates Paclitaxel effect to decrease expression of the reprogramming genes* Nanog, EpCAM, c-Myc, Sox2* and two stemness marker (CD44 and CD133) in the HCC cells *in vitro*. Our results revealed that *GATA5* played an important role in Paclitaxel inhibiting the malignant behaviors of HCC cells by blocking expression of the reprogramming related genes and stemness markers.

## Materials and Methods

### Cell culture

In the experimental study, three human liver cancer cell lines (HLE, Bel7402 and PLC/PRF/5) were selected to test, the HCC cells were purchased from the Institution of Cellular Biology, Shanghai Academy of Life Science, China Academy of Science (Shanghai, China). These cells were cultured in RPMI-1640 medium supplemented with 10% heat-inactivated fetal calf serum (FCS) at 37°C in a humidified atmosphere containing 5% CO_2_. The culture medium was replaced or the cells were passaged according to their growth state after 1-2 days. This study protocol was approved by the Ethical Committee of Hainan Medical College (code: 20170106).

### Construction and transfection of the *GATA5* expression vector

The construct of stable expression vector CDH-*GATA5* was as follows: the full-length human *GATA5* cDNA (residue 1-397, NCBI: NM_080473) was synthesized and amplified by polymerase chain reaction (PCR) using the following primers:

F: 5´-CCGAAGCTTGCCACCATGTACCAGAGCCT-3´

R: 5´-CGGGCGGCCGCCTAGGCCAAGGCCAGCGC-3´.

They were then ligated into the expression vector pCDH-CMV-MCS-EF1-coGFP (Systembio, USA) by the HindIII and NotI restriction enzymes (Takara Bio Inc., China). The expression vector was transfected into HCC cells by Lipofectamine 2000 (Invitrogen, USA). To obtain the stable expression vector CDH-*GATA5*, Puromycin was used to screen the stable cell clones. The transfected Bel7402 and PLC/PFR/5 cells with CDH-*GATA5* were respectively named Bel7402-CDH-*GATA5* and PLC/ PFR/5-CDH-*GATA5*.

### RNA interference

Transfection of siRNA-*GATA5* or its negative control siRNA-scramble into HLE cells was as follows: the cells were seeded into 6-wells plate until they reached 70- 80% confluence. The siRNA-*GATA5* or siRNA-scramble was transfected in each well, in the absence of serum by Lipofectamine 2000. The siRNA-*GATA5* sequence was as follows: 5´-AAAGUCCUCAGGCUCGAAC-3´ (8). The transfected HLE cells with siRNA-*GATA5* were named HLE-siRNA-*GATA5*.

### MTT assay

1.5×10^4^ cells/ml of HLE, Bel7402, PLC/PRF/5, HLEsiRNA- *GATA5*, Bel7402-CDH-*GATA5* and PLC/PRF/5- CDH-*GATA5* were cultured in 96-wells plate in RPMI- 1640 medium supplemented with 10% FCS at 37˚C in a humidified atmosphere of 5% CO_2_ for 48 hours. These cells were refreshed with culture medium containing with 10% FCS and they were next treated with different concentrations (5-20 μg/ml) of Paclitaxel (Sigma- Aldrich, USA) for 24 hours. Effect of Paclitaxel on cell growth was measured by the methylthiazolyldiphenyltetrazolium bromide (MTT) assay. Absorbance of the experimental group was measured by a microplate reader at a wavelength of 490 nm. Growth ratio was calculated using the following formula: growth ratio=(control group A_490_-treated group A_490_)/control group A_490_×100% (14).

### Analyses of the cell morphology, cell death and cellular nucleus

The HLE, Bel7402, PLC/PRF/5, HLE-siRNA-*GATA5*, Bel7402-CDH-*GATA5*, and PLC/PRF/5-CDH-*GATA5* cells were inoculated into a 6-wells plate with the concentration of 2.5×10^4^ cells/ml. Then, the cells were cultured in complete medium, containing 10% FCS for 24 hours and they were refreshed with serum-free medium after 12 hours, followed by treating with 10 μg/ml Paclitaxel for 24 hours (in complete medium, containing 10% FCS). Morphology of the cells was observed under a light microscope. Trypan blue staining was used to observe dead cells by microscopy. The cells were also stained with 4,6-diamidino-2-phenylindole dihydrochloride (DAPI) solution to determine potential changes of the cellular nucleus. The cells were imaged using a microscopy at ×100 magnification. The cellular apoptosis condition was assessed under a microscope, and these criteria were described in the previous reports (14-16).

### Soft agar colony formation assay

Approximately 1000 cells were plated in the 6-wells plate and they were cultured in complete medium containing 20% FCS, mixing with 0.7% soft agar (1:1) to lay the upper layer. Then, the cells were incubated for 14 days at 37˚C. The colonies were photographed and counted using a Nikon inverted microscope (Nikon, Japan) (9).

### Crystal violet staining observation of the colony formation

The cells were transferred into the fresh 6-wells plate Petri-dishes at a concentration of 800 cells/well, followed by growth selection using 400 mg/ml of G418 (Beijing Baiaolaibo Science and Technology Ltd., China). After 14 days of incubation, the cells were fixed with 75% ethanol for 30 minutes. They were subsequently stained with 0.2% crystal violet (Beijing Zhongshan Biotechnology Co., China) for colony visualization and counting (9).

### Flow cytometry

The HLE, Bel7402 and PLC/PRF/5 cells were cultured in RPMI-1640 medium supplemented with 10% FCS at 37°C in a humidified atmosphere of 5% CO_2_. The cells were transfected with siRNA-*GATA5* or CDH-*GATA5* for 48 hours, followed by treatment with Paclitaxel (20 μg/ ml) for 48 hours. Apoptosis of the cells were analyzed by flow cytometer (Thermo Fisher Scientific, China) using the method as described previously (14).

### Cells migration assays

The transwell method was used for observing the cells migration, and it was performed according to the manufacturer’s protocol (Biofavor Biotech, China) and as described previously (17, 18). The HLE, Bel7402 and PLC/PRF/5 cells (1.5×10^4^) were transfected with siRNA*GATA5* or CDH-*GATA5* for 48 hours. The cells were added to the upper chamber, cultured in serum-free RPMI-1640 medium and treated with Paclitaxel (10 μg/ml) for 48 hours, while the lower chamber was filled with 20% FCS. The complete medium was manipulated according to the previously described methods (18). Number of the cells migrated through the wells was quantified by counting five independent fields, using a microscopy with a ×20 objective lens (Olympus Corporation, Japan).

### Western blotting

To evaluate the effect of Paclitaxel on migration-related proteins, reprogramming genes and stemness markers, the cells were transfected with siRNA-*GATA5* or CDH*GATA5* followed by treatment with Paclitaxel (10 μg/ml) for 48 hours. A Western blot analysis of the metastasisassociated proteins MMP2 and MMP9 as well as the reprogramming related genes *Nanog, c-Myc, Sox2,* and *EpCAM* were performed. The experimental method was described previously (14, 19).

### Detection of proteins expression by laser confocal microscopy

Expression of the stemness markers (CD44 and CD133) was observed by laser confocal microscopy after drug screening. HLE, Bel7402 and PLC/PRF/5 cells were transfected with siRNA-*GATA5* or CDH-*GATA5* for 24 hours. They were then inoculated into the laser confocal culture chamber for 12 hours, followed by treating with Paclitaxel (10 μg/ml) for 48 hours. The remaining cells were incubated with rabbit anti-human primary CD44 and CD133 antibodies (Abcam Corp., USA) for 12 hours. Next, they were incubated with fluorescent Alex488 and Alex647 (Beyotime Corporation, China) secondary antibodies for 2 hours. Then, they were washed with PBS and DAPI was added. Expression and localization of CD44 and CD133 were subsequently observed by laser confocal microscopy (Fuji, Japan). The experimental method was described previously (20, 21).

### Statistical analysis

The data are presented as the mean ± SD. Statistical analysis was performed using Student’s t test (for two experimental groups) and F-test (SPSS 11.5 software for Windows, SPSS Inc., USA). The statistical significance was set at P<0.05.

## Results

### GATA5 stimulated Paclitaxel to inhibit the growth of hepatocellular carcinoma cells

**Fig.1:**
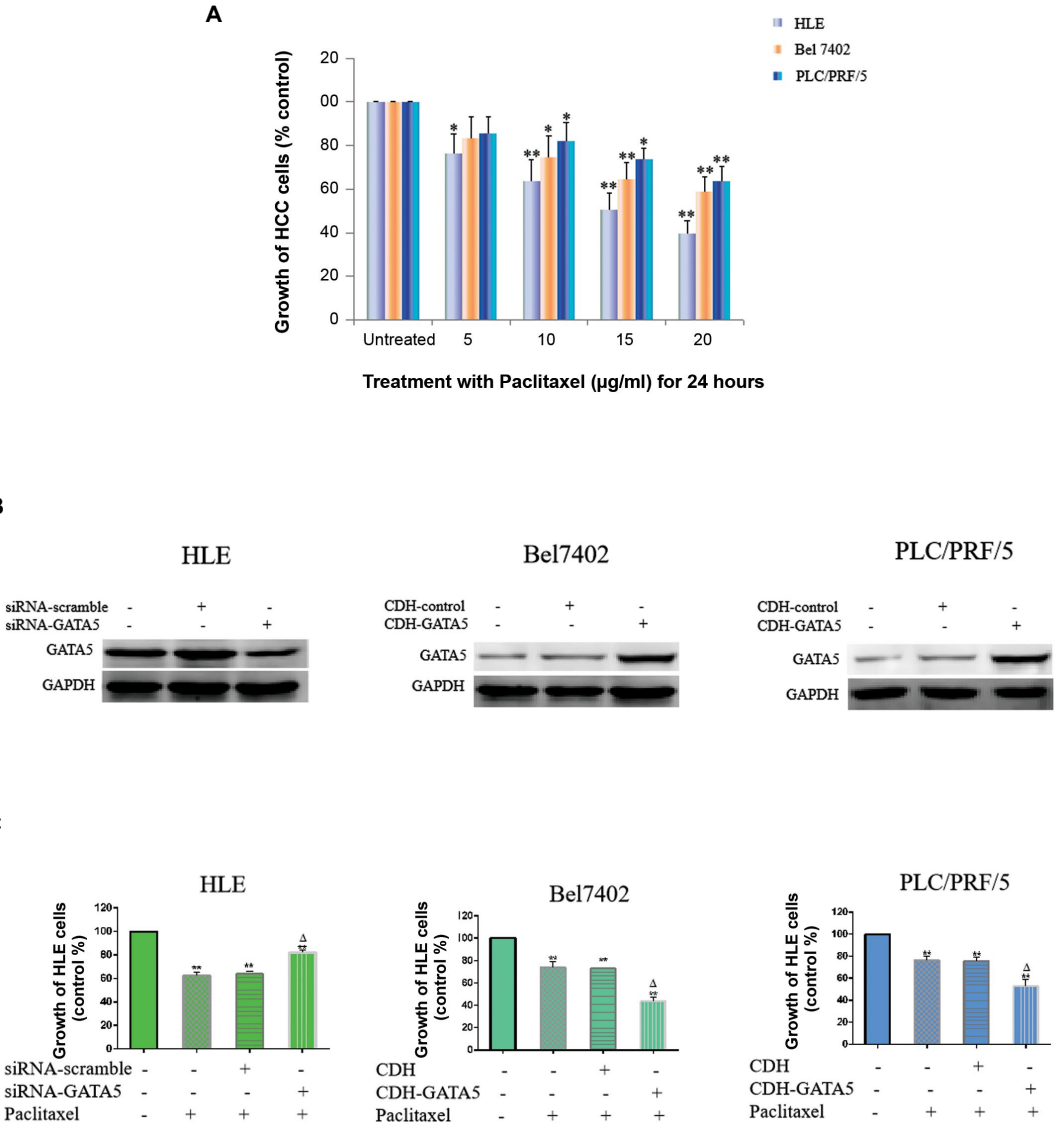
Effect of GATA5 and Paclitaxel on the growth of hepatocellular carcinoma cells (HCC). **A.** HLE, Bel7402 and PLC/PRF/5 cells were treated with different concentrations of Paclitaxel (0, 5, 10, 15 and 20 μg/ml) for 24 hours. Growth of the cells was measured by MTT assay. **B.** Western blotting was carried out to test expression of GATA5 in the HLE cells, Bel7402 and PLC/PRF/5 cells. **C.** The HLE cells were transfected with siRNA-scramble or siRNA-*GATA5* vectors for 24 hours followed by treatment with Paclitaxel (10 μg/ml) for 24 hours. The Bel7402 and PLC/PRF/5 cells were transfected with the CDH empty vectors or CDH-*GATA5* vectors for 24 hours followed by treatment with Paclitaxel (10 μg/ml) for 24 hours. Growth of the cells was measured by an MTT assay. N=6, *; P<0.05, **; P<0.01 versus the control groups, and ^∆^; P<0.01 versus the empty vector groups or the empty vector groups plus the Paclitaxel-treated groups. Three independent experiments were performed for these data.

To investigate the influence of GATA5 on Paclitaxel regulating growth of HCC cells, we first conducted a MTT assay to analyze the influence of different concentrations of Paclitaxel (5-20 μg/ml) on proliferation of HCC cells. When the optimal concentration of paclitaxel was determined as >10 μg/ml, growth of these HCC cells was significantly inhibited (Fig .1A). Then, we used Western blot to test GATA5 expression in the HCC cells. Result showed that the HLE cells had high expression of endogenous GATA5, but the endogenous expression of GATA5 in the Bel7402 and PLC/PRF/5 cells was low (Fig .1B). Thus, we silenced GATA5 expression in the HLE cells by transfecting the cells with siRNA-*GATA5*, and enhanced *GATA5* expression in the Bel7402 and PLC/PRF/5 cells by transfecting with the CDH-*GATA5* expression vector. The MTT result indicated that silencing *GATA5* expression in the HLE cells stimulated growth, which was inhibited by Paclitaxel. Enhancing *GATA5* expression in the Bel7402 and PLC/PRF/5 cells inhibited the growth to a higher degree, synergizing with Paclitaxel (Fig .1C). The results indicated that overexpression of *GATA5* was able to enhance sensitivity of HCC cells to Paclitaxel *in vitro*.

### GATA5 enhanced Paclitaxel to promote apoptosis of hepatocellular carcinoma cells

**Fig.2:**
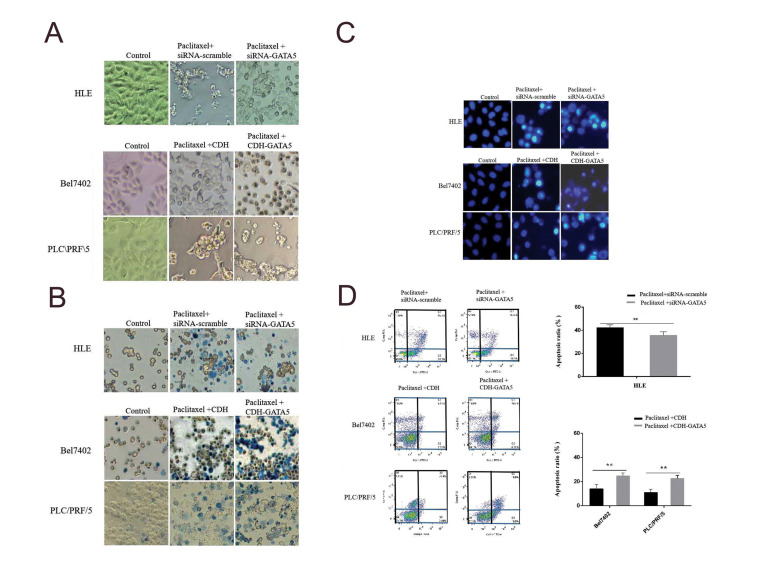
Effect of the GATA5 and Paclitaxel on the apoptosis of hepatocellular carcinoma cells (HCC). The HLE cells were transfected with siRNA-scramble or siRNA-*GATA5* for 24 hours. The Bel7402 and PLC/PRF/5 cell were transfected with the CDH empty vectors or CDH-*GATA5* vectors for 24 hours. Next, these cells were treated with Paclitaxel (20 μg/ml) for 48 hours. Then, apoptosis of the cells was analyzed. **A.** Light microscopy was used to observe morphological changes of the HCC cells (×100). **B.** Trypan blue excluded staining and observations by microscopy (scale bar: ×100). **C. **DAPI staining and morphological observation by microscopy (scale bar: 20 mM). **D.** Flow cytometry was applied to detect apoptosis of HCC cells. The low columnar graph indicates quantity of the cell apoptosis. Three independent experiments were performed for these data. N=5, **; P<0.05 versus siRNA-scramble vectors groups or CDH empty vectors groups.

In this study, we also investigated whether GATA5 was able to enhance Paclitaxel to induce apoptosis of HCC cells by microscopy observations, trypan blue exclude staining, DAPI staining and a flow cytometry analysis. The HCC cells were treated with Paclitaxel for 48 hours followed by transfection with siRNA*GATA5* or CDH-*GATA5* for 48 hours. The results obtained from light microscopy observation (Fig .2A), trypan blue exclude staining observation (Fig .2B) and DAPI staining observation (Fig .2C) showed that Paclitaxel promoted apoptosis of HCC cells. Silencing *GATA5* expression by transfecting with siRNA*GATA5* in the HLE cell antagonized the influence of HCC cell apoptosis, which was induced by Paclitaxel. Increasing expression of GATA5 by transfecting with CDH-*GATA5* in the Bel7402 and PLC/PRF/5 cells could enhance Paclitaxel to induce apoptosis of these HCC cells. The flow cytometry analysis also confirmed that GATA5 was able to promote Paclitaxel-induced apoptosis of the HCC cells. The results showed that number of apoptotic cells was significantly higher in the Paclitaxel+siRNA-scramble group compared to the untreated group. HLE cells were treated with Paclitaxel followed by transfecting with siRNA-*GATA5* to silence expression of this gene (Paclitaxel +siRNA *GATA5* group). The obtained cells displayed that the number of apoptotic cells was reduced compared to Paclitaxel+siRNA-scramble group. Bel7402 and PLC/ PRF/5 cells were treated with Paclitaxel followed by increasing *GATA5* expression by transfecting with CDH-*GATA5* (Paclitaxel+CDH-*GATA5* group). The obtained cells showed that the number of apoptotic cells was increased compared to the transfected cells with the CDH empty vector (Paclitaxel+CDH group, Fig .2D). These results indicated that GATA5 has a trait to enhance the effect of Paclitaxel on inducing apoptosis of HCC cells.

### GATA5 enhanced the effect of Paclitaxel on inhibiting migration and invasion of HCC cells

**Fig.3:**
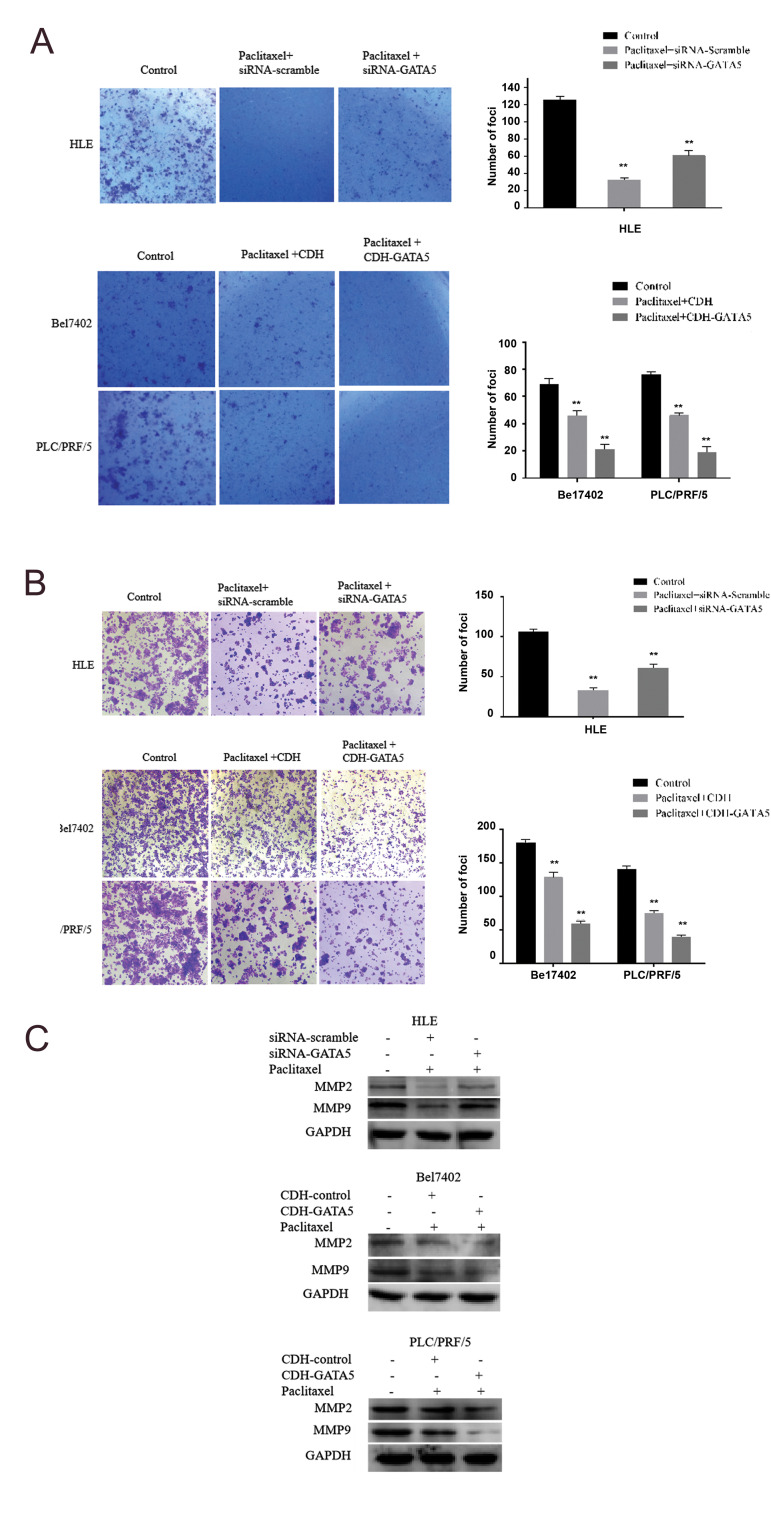
Influence of GATA5 and Paclitaxel on the migration, invasion and expression of the migration-related proteins in hepatocellular carcinoma cells (HCC). The HLE cells were transfected with siRNA-scramble or siRNA-*GATA5*. The Bel7402 and PLC/PRF/5 cell were transfected with the CDH empty vectors or CDH‑*GATA5* vectors for 24 hours followed by treatment with Paclitaxel (10μg/ml) for 48 hours. **A, B.** The migratory and invasive cells were stained with 0.1% crystal violet and they were observed by microscopy. The right columnar graph indicates quantity of the migratory cells (×100). **C. **Western blotting was used to analyze the expression of MMP2 and MMP9 in the HLE, Bel7402 and PLC/PRF/5 cells. Three independent experiments were performed for these data. N=8, **; P<0.05 versus the control group.

In this study, we also investigated whether GATA5 synergizes with Paclitaxel to inhibit HCC migration by a transwell analysis. The microscopic observations showed that after silencing *GATA5* expression by transfecting with siRNA-*GATA5* in the Paclitaxel treated HLE cells (Paclitaxel+siRNA-*GATA5* group), pore transfer capacity of the cells was significantly increased compared to the cells transfected with siRNA-scramble (Paclitaxel+siRNAscramble group). Enhancing *GATA5* expression by transfecting with CDH-*GATA5* in the Paclitaxel-treated Bel7402 and PLC/PRF/5 cells (Paclitaxel+CDH-*GATA5* group), showed that pore migration capacity of the HCC cells was decreased compared to the cells transfected with the CDH empty vector (Paclitaxel+CDH group, Fig .3A, B). These data indicated that GATA5 synergized with Paclitaxel to inhibit HCC cells migration and invasion.

We also assessed the influence of GATA5 on the expression of the metastasis-related factors, MMP2 and MMP9. In the present study, Western blot results indicated that after silencing expression of *GATA5* by transfecting with siRNA-*GATA5* in the Paclitaxeltreated HLE cells (Paclitaxel+siRNA-*GATA5* group), there was a higher expression of MMP2 and MMP9, compared to the cells transfected with siRNA-scramble (Paclitaxel+siRNA-scramble group). Enhancing GATA5 expression by transfecting with CDH-*GATA5* in Bel7402 and PLC/PRF/5 cells (Paclitaxel+CDH-*GATA5* group) revealed a reduced expression of MMP2 and MMP9, compared to the cells transfected with the CDH empty vector (Paclitaxel+CDH group, Fig .3C). These results demonstrated that GATA5 played a role in synergizing with Paclitaxel to down-regulate expression of the metastasis-related factors, MMP2 and MMP9.

### GATA5 increased the effect of Paclitaxel on inhibiting colony formation of hepatocellular carcinoma cells

**Fig.4:**
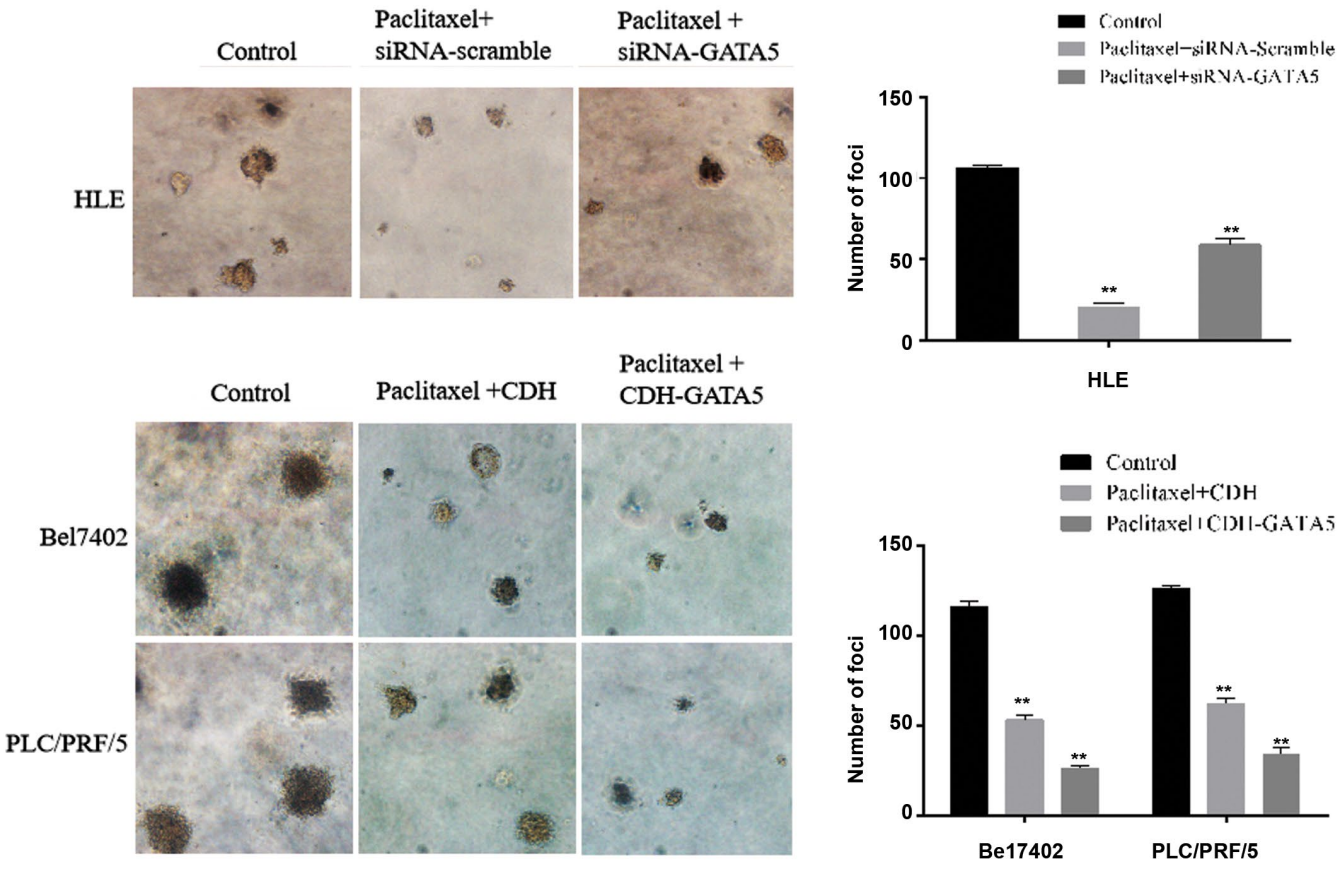
Influence of GATA5 and Paclitaxel on the colony formation of hepatocellular carcinoma cells (HCC). The HLE cells were treated with Paclitaxel and they were transfected with siRNA-scramble vectors or siRNA-*GATA5* vectors. The Bel7402 and PLC/PRF/5 cells were transfected with the CDH empty vectors or CDH.*GATA5* vectors followed by treatment with Paclitaxel (10μg/ml) for 14 days. The colonies were observed by microscopy (×40). The right columnar graph indicates quantity of colony formation. N=6, **; P<0.05 versus the control group.

In addition, we investigated whether GATA5 was able to enhance the effect of Paclitaxel on inhibiting colony formation of the HCC cells. The crystal violet staining and microscopy (×100) observations showed that HLE cells treated with Paclitaxel and transfected with siRNA-scramble (Paclitaxel+siRNA-scramble group) inhibited colony formation. Silencing *GATA5* expression by transfecting with siRNA-*GATA5* in the paclitaxel-treated HLE cells (Paclitaxel+siRNA-*GATA5* group) restored more capacity of colony formation compared to the Paclitaxel+siRNAscramble group. Bel7402 and PLC/PRF/5 cells treated with Paclitaxel and transfected with the empty vector CDH (Paclitaxel+CDH group) showed that number and volume of cellular colony were significantly decreased compared to the control. Increasing expression of GATA5 by transfecting with CDH-*GATA5* in the Bel7402 and PLC/PRF/5 cells (Paclitaxel+CDH-*GATA5* group) demonstrated that number and volume of cellular colony were inhibited compared to the control group and the Paclitaxel+CDH group (Fig .4). These results indicated that GATA5 played a key role in enhancing the effect of Paclitaxel on inhibiting the colony formation of HCC cells.

### GATA5 increased the effect of Paclitaxel on inhibiting expression of the reprogramming genes

**Fig.5:**
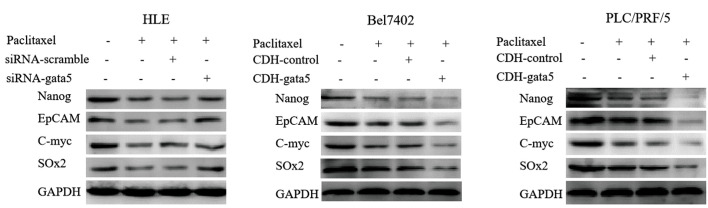
Effect of GATA5 and Paclitaxel on the expression of reprogramming genes in the hepatocellular carcinoma cells (HCC). The HLE cells were transfected with siRNA-scramble vectors or siRNA-*GATA5* vectors. The Bel7402 and PLC/PRF/5 cells were transfected with the CDH empty vectors or CDH-*GATA5* vectors for 24 hours followed by treatment with Paclitaxel (10 μg/ml) for 48 hours. Then, Western blotting was applied to analyze expression of the reprogramming genes:* Nanog, EpCAM, c-Myc* and *Sox2*. The images are representative of three independent experiments.

To investigate how GATA5 mechanistically stimulated Paclitaxel to suppress malignant behaviors of HCC cells, we analyzed expression of the cancer stem cell reprogramming genes, Nanog, EpCAM, c-Myc and Sox2 in the cells by Western blotting. The results indicated that after silencing expression of GATA5 by transfecting the HLE cells with siRNA-*GATA5* (Paclitaxel+siRNA-*GATA5* group) the reprogramming genes were upregulated in comparison with the cells transfected with siRNA-scramble (Paclitaxel+siRNAscramble group). After enhancing expression of GATA5 by transfecting with CDH-*GATA5* in Bel7402 and PLC/PRF/5 cells (Paclitaxel+CDH-*GATA5* group), expression of the reprogramming genes was reduced compared to the cells transfected with the CDH empty vector (Paclitaxel+CDH group, Fig .5). These results indicated that GATA5 enhanced Paclitaxel to inhibit expression of Nanog, EpCAM, c-Myc and Sox2 in HCC cells.

### GATA5 promoted Paclitaxel to inhibit expression of stemness markers, CD44 and CD133 in hepatocellular carcinoma cells

**Fig.6:**
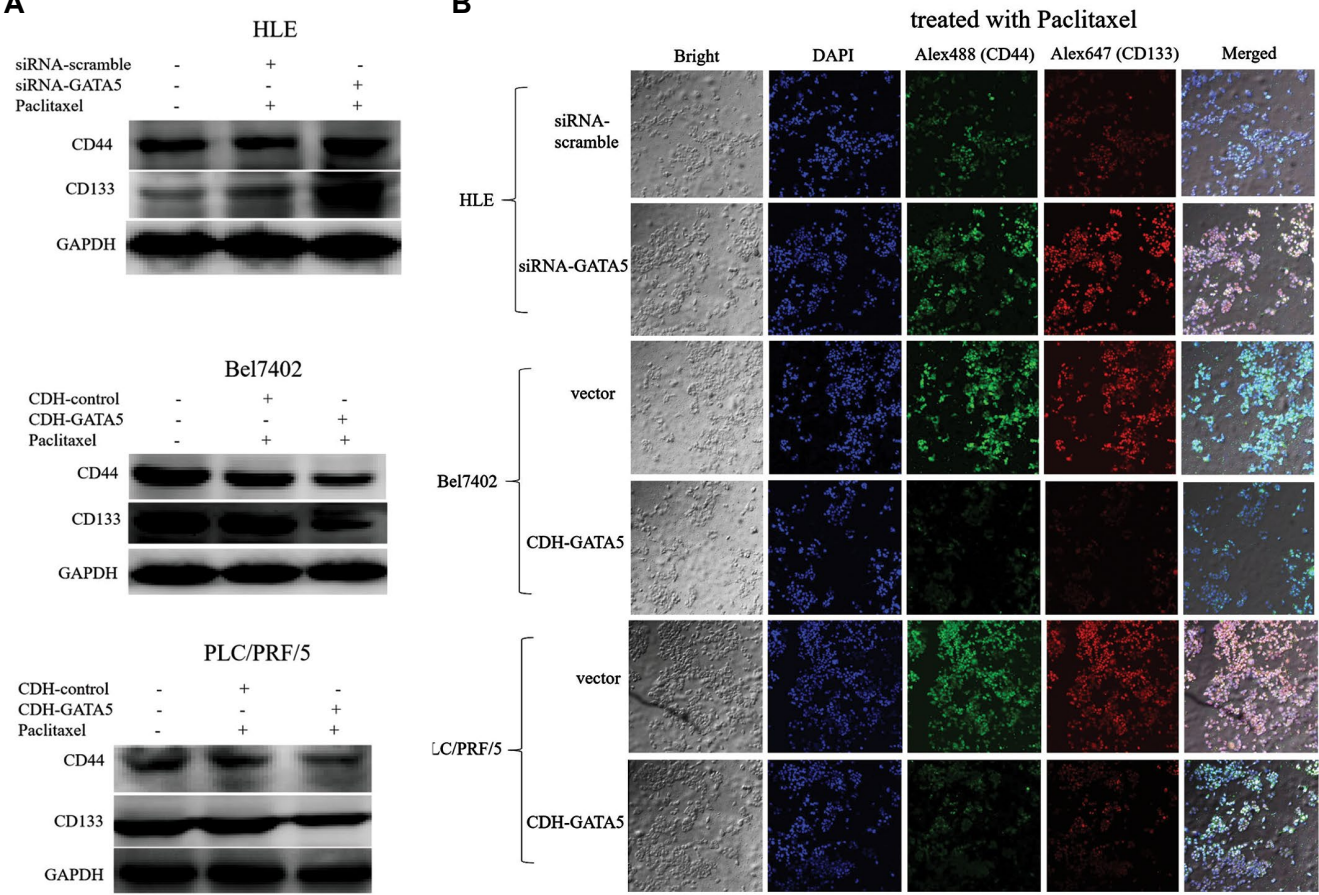
Effect of GATA5 and Paclitaxel on the expression of stemness markers, CD44 and CD133 in the HCC cells. The HLE cells were transfected with siRNAscramble vectors or siRNA-*GATA5* vectors for 24 hours. The Bel7402 and PLC/PRF/5 cells were transfected with the CDH empty vectors or CDH-*GATA5* vectors for 24 hours, and then the cells were treated with Paclitaxel (10 μg/ml) for 48 hours. **A.** Western blotting was used to analyze expressions of CD44 and CD133 in the HCC cells. **B.** The expression and localization of CD44 and CD133 in the HCC cells were visualized by laser confocal microscopy. The nuclei were stained with DAPI (blue). CD44 (green) and CD133 (red) were labeled with Alex488 and Alex647, respectively (×40). Three independent experiments were performed for these data.

The stemness markers CD44 and CD133 play a key role in maintaining the malignancy of HCC. Thus, in this study, we investigated whether GATA5 was able to play a role in stimulating Paclitaxel to suppress expression of CD44 and CD133 in HCC cells. Western blot was performed to assay expression of these proteins and laser confocal microscope observation was applied to detect expression and location of these markers in the HCC cells. The results indicated that silencing expression of *GATA5* (transfected cell with siRNA-*GATA5*) resulted in a higher expression of CD44 and CD133 compared to the HLE cells transfected with siRNA-scramble. After enhancing expression of *GATA5* (transfected cells with CDH-*GATA5*) in the Bel7402 and PLC/PRF/5 cells, expressions of CD44 and CD133 were suppressed, compared to the cells transfected with CDH empty vector (Fig .6A, B). These results showed that GATA5 played a role in promoting Paclitaxel to inhibit expression of the stemness markers, CD44 and CD133 in HCC cells.

## Discussion

Paclitaxel is now widely used as a chemotherapeutic drug for treatment of many types of cancer. It blocks the M/G2 cell cycle and stimulates caspase signal transduction to promote apoptosis in cancer cells (22, 23). Due to the inherent or late acquired drug resistance of liver cancer cells, sensitivity of HCC cells to Paclitaxel is reduced, limiting application of Paclitaxel in the treatment of liver cancer (24, 25). Drug resistance in the liver cancer cells is a crucial problem in clinical treatment. Our study showed that the endogenous expression of GATA5 was higher in the HLE cells than in Bel7402 and PLC/PRF/5 cells. Thus, we silenced expression of *GATA5* in the HLE cells and enhanced expression of *GATA5* in the Bel7402 and PLC/PRF/5 cells. Silencing *GATA5* expression in the HLE cells could antagonize Paclitaxel to inhibit proliferation and stimulate apoptosis of the HCC cells. Inversely, enhancing *GATA5* expression in the Bel7402 and PLC/PRF/5 cells was capable to promote Paclitaxel to inhibit proliferation and stimulate apoptosis of these HCC cells. The results indicated that GATA5 has a trait to inhibit growth and stimulate apoptosis of HCC cells. Additionally, findings suggested that GATA5 was able to increase sensitivity of HCC cells to Paclitaxel.

To further demonstrate whether GATA5 synergized with Paclitaxel to suppress malignant behaviors of HCC cells, we performed HCC cellular colony formation, migration and invasion assays to assess the influence of GATA5 in the HCC cells accompanied by the treatment with Paclitaxel. Colony formation assay indicated that GATA5 synergizes with Paclitaxel to significantly inhibit cellular colony formation in the HCC cells. The cell migration and invasion assay indicated that GATA5 synergized with Paclitaxel to significantly reduce pore migratory capacity of the HCC cells and overexpression of GATA5 enhanced Paclitaxel to inhibit expression of the migrationrelated factors, MMP2 and MMP9. These results further demonstrated that GATA5 promoted Paclitaxel to induce apoptosis of HCC cells. Enhancing expression of GATA5 was able to synergize with Paclitaxel to inhibit HCC cells migration and invasion. GATA5 increased the sensitivity of HCC cells to Paclitaxel which maybe involved in suppressing expression of MMP2 and MMP9.

Cancer stem cells play pivotal role in malignant cells transformation (26, 27). Reprogramming genes, such as *Nanog, KLF4, EpCAM, c-Myc, Sox2* and *p-Oct4* induce the origin of CSCs (10, 11, 21). In the present study, we performed a Western blot analysis to assess influence of GATA5 and Paclitaxel on expression of reprogramming genes in HCC cells. Silencing *GATA5* expression in HLE cells (accompanied with treatment by Paclitaxel) stimulated expression of the reprogramming genes, while enhancing expression of GATA5 in the Bel7402 and PLC/ PRF/5 cells (accompanied with treatment by Paclitaxel) could inhibit expression of the reprogramming genes, such as *Nanog, c-Myc* and *Sox2*. The results indicated that GATA5 played a role in promoting Paclitaxel to inhibit expression of the reprogramming genes in HCC cells, which enhanced sensitivity of the HCC cells to Paclitaxel, and also inhibiting cancer stem cell formation and cancer cells aggressiveness. Previously, we found that hepatitis virus B x protein (HBx) promoted liver cancer stem cell genesis by stimulating expression of alpha fetoprotein (AFP); so that AFP promoted expressions of CD44 and CD133 (21). AFP is a critical molecule to promote malignant transformation of liver cells and inhibit autophagy of HCC cells (28). These results implicated that expression of CD44 and CD133 in liver cancer cells is related to the malignant behaviors. CD44 is a stemness marker involved in cell adhesion and tumor metastasis (12, 29). CD133 is considered a stemness marker of cancer cells which plays a critical role in cancer recurrence (12). Recently, we found that GATA5 played role in inhibiting expression of reprogramming genes in HCC cells (30). In the present study, we also found that GATA5 synergized with Paclitaxel to inhibit expression of the stemness markers CD44 and CD133 in the cancer stem cells. These results further demonstrated that overexpression of GATA5 was able to enhance the effect of Paclitaxel on inhibiting HCC cells malignant behaviors. These mechanisms maybe involved in suppressing the expression of reprogramming genes.

## Conclusion

This is the first report indicating that GATA5 plays a role in promoting Paclitaxel to inhibit the growth, migration, invasion and colony formation of HCC cells, in addition to stimulating apoptosis in these cells. All together, we revealed that in terms of molecular mechanism, GATA5 synergizes with Paclitaxel to inhibit the malignant behaviors of HCC cells which maybe involved in suppressing expression of reprogramming genes and stemness markers. These findings suggest that enhanced expression of GATA5 may be an available strategy for applying Paclitaxel to treat HCC patients.
